# Inappropriate Use of Antibiotics and Its Associated Factors among Urban and Rural Communities of Bahir Dar City Administration, Northwest Ethiopia

**DOI:** 10.1371/journal.pone.0138179

**Published:** 2015-09-17

**Authors:** Endalew Gebeyehu, Laychiluh Bantie, Muluken Azage

**Affiliations:** 1 Department of Pharmacology, College of Medicine and Health Sciences, Bahir Dar University, P.O Box 79, Bahir Dar, Ethiopia; 2 Department of Public Health, College of Medicine and Health Sciences, Bahir Dar University, P.O.Box 79, Bahir Dar, Ethiopia; National Institute of Health, ITALY

## Abstract

**Background:**

Inappropriate use of antibiotics in the community plays a role in the emergence and spread of bacteria resistant to antibiotics which threatens human health significantly. The present study was designed to determine inappropriate use of antibiotics and its associated factors among urban and rural communities of Bahir Dar city administration.

**Methods:**

A comparative cross sectional study design was conducted in urban and rural kebeles of Bahir Dar city administration from February 1 to March 28, 2014. A total of 1082 participants included in the study using a systematic random sampling technique. Data was collected using pre-tested and structured questionnaire. Data was coded and entered into SPSSS version 16 for statistical analysis. Bivariate and multivariate logistic regression model were used to identify factors associated with inappropriate use of antibiotics.

**Results:**

Inappropriate use of antibiotics was 30.9% without significant difference between urban (33.1%) and rural (29.2%) communities. From the inappropriate antibiotic use practice, self-medication was 18.0% and the remaining (12.9%) was for family member medication. Respiratory tract symptoms (74.6%), diarrhea (74.4%), and physical injury/wound (64.3%) were the three main reasons that the communities had used antibiotics inappropriately. Factors associated with inappropriate use of antibiotics were low educational status, younger age, unsatisfaction with the health care services, engagement with a job, and low knowledge on the use of antibiotic preparations of human to animals.

**Conclusions:**

Inappropriate use of antibiotic exists in the study area with no significant difference between urban and rural communities. The study indicated an insight on what factors that intervention should be made to reduce inappropriate use of antibiotics in the community. Interventions that consider age groups, educational status, common health problems and their jobs together with improvement of health care services should be areas of focus to reduce inappropriate use of antibiotics.

## Introduction

Antibiotics are the most commonly prescribed drugs in many developed [1–3] and developing countries [4, 5]. A systematic review showed that inappropriate use of antibiotics was common especially in the developing countries with poor health care systems [6]. Inappropriate use of antibiotics can result in bacteria resistant to antibiotics in the community [7–10]. The acceleration of antibiotic resistance and the decline in the development of new antibiotics to combat the problem has created a significant public health challenges to health policy makers, health care workers, and the population around the world [1, 11].

Studies found that inappropriate use of antibiotics was associated with different factors: culture [12, 13], gender [14, 15], educational status [15–18], residency [19], marital status [15], age [20–25], number of children [19], health insurance [15] and unstatisfaction with the health care services [17, 26–28], and storing antibiotics at home [19, 29].

World Health Organization (WHO) estimated that 80% of antibiotics is used in the community, of which about 20–50% is used inappropriately [11]. As a result, WHO recommended involvement of the community in tackling of antibiotic resistance through improving access to medical services, reducing unnecessary use of antibiotics, taking a full course of treatment, not sharing medications with other people, and not keeping part of the course for another occasion [30]. There is variation in antibiotic use among and within counties related with several factors [6]. Thus, to draw effective intervention requires exploration of factor associated with inappropriate antibiotic use in the community.

In Ethiopia, accessibility of health care services has been improved in the past two decades. According to Drug Administration and Control Authority, there are indications on inappropriate use of antibiotics in the country [31]. However, the extent inappropriate use of antibiotics and its associated factors has not yet been explored. Therefore, the aim of this study was to determine inappropriate use of antibiotic and its associated factors among urban and rural communities in Bahir Dar city administration, Northwest Ethiopia.

## Materials and Methods

### Study design and period

A comparative cross sectional study design was employed from February 1 to March 28, 2014 to determine inappropriate use of antibiotic and its associated factors among urban and rural communities in Bahir Dar city administration.

### Study area and population

Bahir Dar city administration, the study area, is one of the three city administrations in Amhara Regional State located at Northwest of Ethiopia. The city administration includes nine urban and nine rural kebeles. Based on the projection of the 2007 national census, the total population of Bahir Dar city administration in 2014 was 284020 of these 134818 were males whereas 149202 were females. Of the total population 226713 were living in urban kebeles and 57307 were living in rural kebeles [32]. Individuals living in the city administration during the study period were the study population. Households lived in the past six months were included in the study. Respondants with health care profession and those with communication defects (listening and speaking of the local language) were excuded in the study.

### Study Variables

Outcome variable (inappropriate use of antibiotics) and explanatory variables (sex, place of residence, age, number of family, family monthly income, education status, occupation, marital status, level of healthcare service satisfaction, knowledge on antibiotics use).

### Sample size determination and sampling technique

Two population proportion formula was used to determine the required sample size of the study using Epi Info 3.5.2 by considering the following assumptions: two comparison groups [urban (n_1_) and rural (n_2_) population with 1:2 urban to rural ratio], P_1_ (50%) [assumed practice of inappropriate use of antibiotics among urban community since there was no previous study], Z1-α/2 (1.96), 80% power, 15% difference between urban and rural (P2 = 35%), 2 design effect and 15% non-response rate. Based on these assumptions, the total sample size was 1101 (367 urban and 734 rural households). By considering the difference between communities related to antibiotic use, multistage stratified random sampling technique was used to select households in rural and urban kebeles. To get a representative sample, three urban and three rural kebeles were selected using simple random sampling technique. In the selected kebeles, systematic random sampling technique was used to select study participants.

### Data collection tool

Pre-tested and structured questionnaire was used to collect data (S1 File). The questionnaire comprises of socio-demographic variables of the study participants, and knowledge and use of antibiotics. Data collectors hold strips of the antibiotics with them to show for respondents whether they can able to differentiate and select the type of antibiotics that they had been using in the last one year.

### Operational definition

Inappropriate use of antibiotics means use of antibiotics for self-medication and/or medication of family members (family medication) without prescription from health professionals, receiving antibiotics from anybody else and/or use of leftover drugs and/or use of prescribed antibiotics for any purpose other than prescribed for.

### Data quality management

Pre-test of the questionnaire was done in another place in the study area to ensure whether the study participants understood what the investigators intended to know and some modification of questions were made. Data collectors were given training and supervised daily by principle investigators to collect quality data.

### Data analysis procedures

Data were entered and analyzed using SPSS statistical package version 21.0 (S2 File). Descriptive statistics such as percentages, means and standard deviations were used to describe data. Chi-square was used to assess urban-rural differences in antibiotic use. Bivariate and multivariable logistic regressions were used to identify predictors of inappropriate use of antibiotics. The Hosmer—lemeshow test was checked to assess the model fitted to conduct logistic regression. Those variables with a p-value in bivariate 0.02 were retained for multivariable logistic regression. A multi-collinearity test was done and variables with variance inflation factors (VIF) of greater than 10 were excluded from the multivariable regression model. Backward stepwise logistic regression model was used during multivariable logistic regression to control confounding effect. Odds ratio with 95% confidence intervals was calculated for each of independent variables using P-value < 0.05 was considered as level of significance.

### Ethical considerations

The study was conducted after obtaining ethical approval from Research Ethics Committee of College of Medicine and Health Sciences, Bahir Dar University. The committee provided ethical approval after assessing informed verbal consent submitted with all components of the research protocol. The verbal consent was included on the front page of the questionnaire below briefing statements of the study. After data collectors read the briefing statements of the study to the participants, the study participant willingness/unwillingness was confirmed by marking their yes/no response. When the participant confirmed his/her willingness, data collection was carried out. Confidentiality and privacy were insured for information collected from study participants by recording data anonymously.

## Results

A total of 1082 households, 362 urban and 719 rural, participated in the study. The response rate was 98.3%. The mean family household size of the study population was 3.8 (±2.0 SD) in urban and 4.0 (±2.1 SD) in rural communities. The mean age of the study participants was 34.1 (±12.9 SD) in urban and 34.5 (±11.4 SD) in rural households. More than three-quarters of both urban (77.1%) and rural (75.0%) participants were females. The mean family income of households in urban and rural was 1280 and 249 Ethiopian Birr per month respectively. More than one quarter (25.4%) urban and 10.8% rural participants completed secondary school, whereas 20.4% urban and 52.0% rural participants were unable to read and write. Nearly 60% urban and 73.3% rural participants were engaged with a regular job. Nearly three-quarters (71.5%) and 68.8% of the participants in urban and rural communities were married respectively (Table 1).

**Table 1:** Socio-demographic Characteristics of Urban and Rural Study Participants Bahir Dar City Administration, February 1 to March 28, 2014.

Variables	Urban n(%)	Rural n(%)	Total
Sex			
Male	83(22.9)	180(25.0)	263(24.3)
Female	279(77.1)	540(75.0)	819(75.7)
Age			
<25	87(24.0)	152(21.0)	239(22.1)
25–34	130(35.9)	215(29.9)	345(31.9)
35–44	74(20.4)	220(30.6)	294(27.2)
45–54	33(9.1)	92(12.8)	125(11.6)
>54	38(10.5)	41(5.7)	79(7.3)
Number of family size			
1–2	97(26.8)	187(26.0)	284(26.2)
3–5	196(54.1)	343(47.7)	539(49.8)
>5	69(19.1)	190(26.4)	259(23.9)
Family monthly income			
≤1000	123(34.0)	111(15.4)	234(21.6)
1001–2000	71(19.6)	560(77.9)	631(58.3)
2001–3000	116(32.0)	42(5.8)	158(14.6)
3001–4000	39(10.8)	4(0.6)	43(4.0)
>4000	13(3.6)	3(0.4)	16(1.5)
Education status			
Unable to read and write	74(20.4)	374(52.0)	448(41.4)
Able to read and write	37(10.2)	44(6.1)	81(7.5)
Completed primary school	85(23.5)	189(26.3)	274(25.3)
Completed secondary school	92(25.4)	78(10.8)	170(15.8)
Completed college	74(20.4)	35(4.9)	109(10.1)
Job engagement			
Engaged with regular job	209(57.7)	528(73.3)	737(69.1)
Without regular job	153(42.3)	192(26.7)	345(31.9)
Marital status			
Single	80(22.1)	114(15.8)	195(18.0)
Married	259(71.5)	495(68.8)	754(69.7)
Divorced /Widowed	23(6.4)	111(15.4)	134(12.4)

Of the participants, 97.4% in urban and 98.6% in rural were able to differentiate one or more of the antibiotics displayed to them. The incorrect response rate of participants on the use individual antibiotics was: tetracycline, 76.2% in urban and 78.3% in rural; amoxicillin, 68% in urban and 77.1% in rural; and ciprofloxacin, 80.4% in urban and 92.5% in rural communities. The incorrect response rate of urban and rural communities on the use of antibiotic preparations of humans to treat animals was 27.1% and 16.0%, respectively (Table 2).

**Table 2:** Knowledge on Antibiotics among Urban and Rural Study Participants in Bahir Dar City Administration, February 1 to March 28, 2014.

Variables	Urban n(%)	Rural n(%)	Total
Frequency of visiting health care institution in the past one year			
Once	84(23.2)	145(20.2)	229(21.2)
Twice	52(14.4)	30(4.2)	82(7.6)
Three	21(5.8)	24(3.3)	45(4.2)
Four and above	31(8.6)	14(1.9)	45(4.2)
Never	174(48.1)	507(70.5)	681(62.9)
Level of healthcare service satisfaction			
Satisfied	151(41.7)	179(24.9)	330(30.5)
Unsatisfied	211(58.3)	541(75.1)	752(69.5)
Ability to differentiate displayed antibiotics			
Unable to differentiate	9(2.5)	12(1.7)	21(1.9)
Able to differentiate one	166(45.9)	493(68.5)	659(60.9)
Able to differentiate two	103(28.5)	151(21.0)	254(23.5)
Able to differentiate three	52(14.4)	49(6.8)	101(9.3)
Able to differentiate four and above	32(8.3)	15(2.1)	47(4.3)
Can tetracycline cure all diseases?			
Correct response	86(23.8)	156(21.7)	242(22.4)
Incorrect response	276(76.2)	564(78.3)	840(77.6)
Can amoxicillin cure common cold?			
Correct response	116(32.0)	165(22.9)	281(26.0)
Incorrect response	246(68.0)	555(77.1)	801(74.0)
Can ciprofloxacin cure all types of diarrhea?			
Correct response	71(19.6)	26(3.6)	97(9.0)
Incorrect response	291(80.4)	694(92.5)	985(91.0)
Can antibiotic preparations of humans used to animals?			
Correct response	264(72.9)	605(84.0)	869(80.3)
Incorrect response	98(27.1)	115(16.0)	213(19.7)

Nearly 36% of all participants, 46.4% of urban and 30.6% of rural, had taken antibiotics in the past one year prior to the study period. Amoxicillin was the most commonly used antibiotic in both urban (67.3%) and rural (62.3%) communities followed by ampicillin 10.1% in urban and 11.8% in rural. The overall proportion of inappropriate use of antibiotics in study communities was 30.9% (120/388), whereas inappropriate use in urban and rural areas were 33.1% (56/169) and 29.2%(64/219), respectively. Inappropriate use of antibiotics in urban and rural areas did not show a statistical significant difference. Among respondents who used antibiotics in the last one year, the proportion of self-medication was 18.0% (70/388) and the remaining 12.9%(50/388) was used for family medication. Self-medication in urban and rural were 16.0% (27/169) and 19.6% (43/219), respectively and did not show a statistical significant difference. Use of antibiotics to a family member in urban and rural was 17.2% (29/169) and 9.6% (21/219), respectively, and there was a significant difference between them (X^2^ = 4.87; p-value 0.027) (Table 3). Overall self-medication practice in the general community was 18% (70/388). More than one-quarter (25.6%) urban and 28.6% rural participants responded that they had discontinued the administration of antibiotics once their symptoms subside. The three most commonly reported health problems to which antibiotics had been sought in both urban and rural communities were respiratory tract symptoms (30.2%), physical injury/wound (14.7%) and diarrhea (11.3%) (Table 4). Respiratory tract symptoms (74.6%), diarrhea (74.4%), and physical injury/wound (64.3%) were the three most common health problems to which inappropriate use of antibiotics in the communities.

**Table 3:** Comparison of antibiotic use in urban and rural areas in Bahir Dar City Administration, February 1 to March 28, 2014.

Variable	Urban	Rural	X^2^	p-value
Inappropriate use			0.683	0.408
Yes	56	64		
No	113	155		
Self-medication			0.863	0353
Yes	27	43		
No	142	176		
Family member medication			4.870	0.027
Yes	29	21		
No	140	198		

**Table 4:** Extent of inappropriate antibiotic use among urban and rural study participants in Bahir Dar city administration, February to March 2014.

Variables	Urban n(%)	Rural n(%)	Total
Use of antibiotics in the last one year (N = 1082)			
Yes	169(46.7)	219(30.4)	388(35.9)
No	193(53.3)	501(69.6)	694(64.1)
Commonly utilized antibiotics n = 388			
Amoxicillin	113(66.9)	137(62.6)	250(64.4)
Ampicillin	17(10.1)	26(11.9)	43(11.1)
Tetracycline	11(6.5)	15(6.8)	26(6.7)
Ciprofloxacin	9(5.3)	19(8.7)	28(7.2)
Chloramphilicol	6(3.6)	7(3.2)	13(3.4)
Doxycycline	7(4.1)	8(3.7)	15(3.9)
Metrindaloze	5(3.0)	4(1.8)	9(2.3)
Others^a^	1(0.6)	3(1.4)	4(1.0)
Family member(s) to whom antibiotics were used (n = 388)			
To my self	81(50.9)	154(71.3)	235(62.7)
To other family member	78(49.1)	62(28.7)	140(37.3)
Source of used antibiotic (n = 388)			
Prescribed by health care professional	112(67.3)	155(70.5)	267(68.8)
Bought from pharmacy without prescription	41(24.4)	19(8.6)	60(15.5)
Shared from family member or neighbor	16(8.3)	45(21.0)	61(15.7)
Did you discontinue therapy once your symptoms subside? (n = 388)			
Yes	43(25.6)	63(28.6)	106(27.3)
No	126(74.6)	156(71.2)	282(72.7)
For what health problem(s) that the antibiotics were used? (n = 388)			
Respiratory tract symptoms	72(4.9)	45(20.4)	117(30.2)
Diarrhea	18(10.7)	26(11.9)	44(11.3)
Injury/wound	22(13.1)	35(16.0)	57(14.7)
Urinary tract symptoms	5(3.0)	4(1.8)	9(2.3)
Colic	6(3.6)	9(4.1)	15(3.9)
Headache	7(4.1)	17(7.8)	24(6.2)
I could not remember	39(23.1)	83(37.9)	122(31.4)

^a^ Cortimoxazole and Cloxacillin

### Factors associated with inappropriate use of antibiotics

In bi-variate logistic regression analysis, satisfaction with health care services, and knowledge of participants on antibiotic preparations of humans to treat animals were factors associated with inappropriate use of antibiotics. In multivariable logistic regression model, age, educational status, engaged with a regular job, satisfaction with health care services and knowledge of participants on antibiotic preparations of humans to treat animals were statistically significant association with inappropriate use of antibiotics. Those whose age less than 25 and 25 to 34 years were 4.45 [AOR: 4.45; 95%CI (1.54, 12.85)] and 2.73 [AOR: 2.73; 95%CI (1.03, 7.24)] times more likely to use antibiotics inappropriately than those whose age is greater than 54 years. Those who can unable to read and write were 4.21 [AOR: 4.21; 95%CI: (1.47–12.07)] times more likely to use antibiotics inappropriately than those who completed college and above. Those who had engaged themselves in regular jobs were 1.94 [AOR: 1.94; 95%CI: (1.13–3.32)] times more likely to practice inappropriate use of antibiotics than their counterparts. Those who unsatisfied with the health care services were 3.51 times [AOR: 3.51; 95%CI (2.14, 5.78)] more likely to practice inappropriate use of antibiotics than those satisfied with the health care services. Those who had poor knowledge on the use of the antibiotic preparations of humans to treat animals were 3.30 times [AOR: 3.30; 95%CI (1.82, 6.01)] more likely to practice inappropriate use of antibiotics that their counterparts (Table 5).

**Table 5:** Logistic Regression Analysis of Factors associated with inappropriate antibiotic use in Bahir Dar City Administration, February 1 to March 28, 2014 (n = 388).

Variables	Antibiotic use		COR (95%CI)	AOR(95%CI)
	Inappr.	Appr.		
Sex				
Male	30	74	1.00	
Female	90	194	1.14(0.70–1.87)	
Place of residence				
Urban	56	113	1.20(0.78–1.85)	
Rural	64	155	1.00	
Age				
<25	31	65	1.10(0.47–2.58)	4.45(1.54–12.85)*
25–34	40	76	1.21(0.53–2.79)	2.73(1.03–7.24)*
35–44	24	70	0.79(0.33–1.89)	1.20(0.45–3.23)
45–54	15	34	1.02(0.40–2.65)	1.25(0.42–3.74)
>54	10	23	1.00	1.00
Number of family				
1–2	33	66	1.00	
3–5	56	139	0.81(0.48–1.36)	
>5	31	63	0.98(0.54–1.79)	
Family monthly income				
<1000	30	78	1.00	
1000–2000	59	118	1.30(0.77–2.20)	
2001–3000	26	51	1.33(0.70–2.50)	
3001–4000	3	16	0.49(0.13–1.79)	
>4000	2	5	1.04(0.19–5.65)	
Education status				
Unable to read and write	56	81	1.73(0.77–3.88)	4.21(1.47–12.07)*
Able to read and write	10	22	1.14(0.40–3.40)	2.17(0.63–7.44)
Completed primary school	30	83	0.90(0.39–2.10)	1.91 (0.71–5.14)
Completed secondary school	14	57	0.61(0.24–1.57)	1.01(0.34–2.94)
Completed college	10	25	1.00	1.00
Job engagement				
Engaged with regular job	68	143	1.14(0.74–1.76)	1.94(1.13–3.32)*
Without regular job	52	125	1.00	1.00
Marital status				
Married	88	193	1.00	
Single	22	42	1.15(0.65–2.04)	
Divorced/Widowed	10	33	0.67(0.31–1.41)	
Level of healthcare service satisfaction				
Satisfied	54	199	1.00	1.00
Unsatisfied	66	69	3.53(2.24–5.54)**	3.51(2.14–5.78)**
Differentiate the displayed antibiotics you have been used before?				
Unable to differentiate	3	5	1.00	
Able to differentiate one	50	118	0.71 (0.16–3.07)	
Able to differentiate two	38	82	0.77(0.18–3.40)	
Able to differentiate three	21	39	0.90(0.20–4.13)	
Able to differentiate four and above	8	24	0.57(0.11–2.86)	
Can tetracycline cure all diseases?				
Correct response	21	51	1.00	
Incorrect response	99	217	1.11(0.63–1.94)	
Can amoxicillin cure common cold?				
Correct response	32	96	1.00	
Incorrect response	88	172	1.54(0.95–2.47)	
Can ciprofloxacin cure all types of diarrhea?				
Correct response	13	32	1.00	
Incorrect response	107	236	1.12(0.56–2.21)	
Can antibiotic preparations of humans used to animals?				
Correct response	81	232	1.00	1.00
Incorrect response	39	36	3.10(1.85–5.21)*	3.30(1.82–6.01)**

** = P value < 0.01,

* = P value < 0.05; Inappr. = inappropriate; Appr. = Appropriate

## Discussion

The inappropriate use of antibiotics has become a culture of the various communities in different countries which favored for the emergence of resistance to most antibiotics used globally [12, 13, 33–35]. In this study, the proportion of inappropriate use of antibiotics in urban (33.1%) and rural (29.2%) areas did not show significant difference. In addition, self-medication practice did not show significant difference between urban (16.0%) and rural (19.6%). The possible reason could be close proximity of rural community to urban community that may allow the exchange of information. However, family member medication practice between urban (17.2%) and rural (9.6%) areas showed significant difference. These differences may arise from better recall of antibiotics given to themselves than to family members from previous healthcare services in rural community since most are unable to read and write compared to urban communities. In support of this difference, there are studies that show high rates of family member medication in urban [29] than rural area [36, 37].

The overall inappropriate use of antibiotics in this study was 30.9% of which 18% was used for self medication and 12.9% was used for treament of family member. Self-medication finding in this study is comparable with studies done on self-medication in Portugal (19%) [25] and Euro-Mediterranean region (19.1%) [38] but it is lower than that in in northwest Ethiopia (27.5%) [39], Egypt (23.3%) [40], Syrian [34], Greece [41], and Spain [8]. However it is higher than a study done in south Ethiopia (14.5%) [42], Jordan [43], Kuwait [44], and Italy [45]. Family medication study in china found that 59.4% which is higher than this finding [37]. The above difference may be due to level of awareness of the community on antibiotics, difference in education status, socio-economic status, the access to modern health facilities, and cultural preferences and beliefs of the study participants [12, 13].

The three most commonly used antibiotics were Amoxicillin (64.4%), Ampicillin (11.1%) and Ciprofloxacin (7.2%). On top of that, Amoxicillin (34.9%), Ampicillin (17.9%), and Ciprofloxacin (26.8%) were antibiotics used inappropriately in the communities. This result is comparable to the study done in Uganda [46], Indonesia [47], Guatemala [48], Greek [14, 41], and Mongolia [29], which reported that Amoxicillin was the most common antibiotics used by the study participants. Health facility studies in Ethiopia showed that Amoxicillin was most commonly prescribed antibiotic from which its common use in community could arise [49–51].

The effectiveness of antibiotics is threatened by antimicrobial resistance that can arise from discontinuation of the full course of treatment [6]. Studies found that the reasons for discontinuation of antibiotics may be due to lack of knowledge and awareness regarding antibiotic use [21, 52, 53]. In this study, 27% of the respondents discontinued their antibiotics once the symptoms subside. This finding is comparable with the study done in China [54]. However, it is lower than other studies done elsewhere [14, 52, 53] and a meta analysis result of different regions [6]. These differences may be due to overall proportion of antibiotics use in the study participants, the belief of participants on discontinuation on symptoms subsiding, the nature of health problems, knowledge on antibiotics use and extent of health professional advice.

The most common health problems reported in this study were respiratory tract symptoms (30.2%), injury/wound (14.7%), and diarrhea (11.3%). Parallel with this, respiratory tract symptoms (74.6%), diarrhea (74.4%), and physical injury/wound (64.3%) were the three main reasons that the communities had used antibiotics inappropriately. The above findings are similar with studies done in Ethiopia [39], and Euro-Mediterranean region [38].

This study identified individuals in demographic groups who practiced inappropriate use of antibiotics. These groups included individuals with lower educational status and those in younger age groups. Study participants who can unable to read and write were 4 times more likely to use antibiotics inappropriately than those who completed college and above. Other studies have also found similar results that lower educational status was associated with inappropriate use of antibiotics, which may be the result of insufficient knowledge to obtain relevant information on use of antibiotics [17, 18, 34]. Studies done elsewhere indicated that the youngest age groups were higher users of antibiotics inappropriately than the older age groups [24, 25, 34] which is similar with the result of this study. This age related difference in inappropriate use of antibiotics may be due to the extent of experience acquired in living within the community.

Engagement with regular job was one of the predictors of inappropriate use of antibiotics in this study, which is similar to other studies [17, 21, 55, 56]. The possible explanation could be due to lack of time to visit health care facilities during working hours, which may enforce to obtain antibiotics without prescription [55]. The other possible reason could be having pocket money that might encourage them to buy antibiotics when they perceive sign and symptoms of health problems.

Unsatisfaction with health care services was one of the predictors of inappropriate use of antibiotics in this study, which is similar to studies done in other countries [17, 26–28]. Unsatisfaction with health care services may discourage users to seek services from health care facilities and encourage them to look for other options elsewhere has been documented as possible reasons [57]. Poor level of knowledge on the use of the antibiotic preparations of humans to treat animals was also identified as a factor associated with inappropriate use of antibiotics. This might be due to insufficient knowledge and/or belief of participants on the discriminate use of antibiotic preparations between humans and animals.

Unlike previous studies, this study explored factors associated with both self-medication and family member medication. One of the limitations of this study was the use of a cross-sectional design could not able to establish cause and effect relationship between factors and outcome variable. In addition, the study design could not examine the change of inappropriate use of antibiotics in the communities over time. Furthermore, despite the use of pre-tested and structured questionnaire, and showing strips of antibiotics to participants, use of some medical terminology was an inevitably which might be difficult to understood illnesses and names of antibiotics easily.

## Conclusions

Inappropriate use of antibiotic exists in the study area with no significant difference between urban and rural communities. Low education status, younger age, unsatisfaction with health care services, engagement with job, and poor knowledge on the use of antibiotic preparations of human to treat animals were factors associated with inappropriate use of antibiotics. The study indicated an insight on what factors that intervention should be made to reduce inappropriate use of antibiotics in the community. Interventions that consider age groups, educational status, common health problems and their jobs together with improvement of health care services should be areas of focus to reduce inappropriate use of antibiotics.

## Supporting Information

S1 File Questionnaire. (DOCX)

S2 File Data on inappropriate antibiotic use. (SAV)
