# Supplementary material for: Inappropriate Use of Antibiotics and Its Associated Factors among Urban and Rural Communities of Bahir Dar City Administration, Northwest Ethiopia
Source: PLoS One. 2015 Sep 17;10(9):e0138179. doi: 10.1371/journal.pone.0138179 (PMC4574735; doi:10.1371/journal.pone.0138179)
Supplement: S1 File — (DOCX) [file pone.0138179.s001.docx]

**Bahir Dar University**

**College of Medicine and Health Sciences**

**Consent Information sheet**

My name is ………………………………….. I am here on behalf of Bahir Dar University research group staffs. They are conducting a research on ‘Inappropriate Use of Antibiotics and Its Associated Factors among Urban and Rural Communities of Bahir Dar City Administration, Northwest Ethiopia.**’** Their research project was approved by the Research Ethics comets from Bahir Dar university and Bahir Dar city administrators to conduct this study.

You are selected randomly to participate in this study. Your participation is purely based on your willingness.You have the right to choose not to take part in this study. If you choose to take part, you have the right to stop at any time. If you are willing to participate or refuse or decide to withdraw later, you will not be subjected to any ill-treatment.

If you agree to participate in the study, you will be asked to answer some questions about yourself and your family, your knowledge antibiotics and their use. The interview with you will take about 20 minutes. The study will explore about antibiotic use and related factors. It can also provide baseline data for policy makers and other researchers for further improvements of antibiotic use. The information that you provide will be kept confidential by using only code numbers and locking the data. Do not give your name. No one will have access to the non coded data except the principal investigator and the data will not be used for purposes other than the study. Your willingness and active participation are very important for the success of this study.

Based on the understanding of the information I gave you, are you willing to participate in this study?

1. Yes
2. No

**Part I** **Socio demographic factors of respondent**

| **Sr. No** | **Questions** | **Choice Answers** |
| --- | --- | --- |
| 101 | Sex | 1. Male 2. Female |
| 102 | Age | in years:__________ |
| 103 | Family size | In number________ |
| 104 | Residence | 1. Rural 2. Urban |
| 105 | Marital status | 1. Single 2. Married 3. Divorced 4. Widowed 5. Separated |
| 106 | Religion | 1. Orthodox 2. Protestant 3. Muslim 4. Other, specify__________ |
| 107 | What is your occupation? | 1. Engaged in regular work 2. No regular work |
| 108 | Educational Status | 1. Unable to read and write 2. Read and write 3. Primary education(1-8) con 4. Sedary education (9-12) 5. College and above |
| 109 | Average monthly family income | in birr___________ |

**Part II:** **Knowledge of respondents on antibiotics**

| **Sr. No** | **Questions** | **Choice Answers** |
| --- | --- | --- |
| 201 | How often do you visit healthcare facilities per year? | 1. Not at all 2. Once 3. Twice 4. Three times 5. Four times 6. >5 times |
| 202 | What is the level of satisfaction with the medical services provided by health professionals? | 1. Satisfied 2. Unsatisfied |
| 203 | Which drug(s) you know?  Can you mention its (their) importance? | 1. Amoxicillin 2. Ampicillin 3. Tetracycline 4. Ciprofloxacin 5. Chloramphenicol 6. Metronidazole 7. Doxycycline 8. Other specify_______ 9. No one |
| 205 | What is the source of your information? | 1. Relatives 2. Friends 3. Society 4. Health professional 5. Radio/TV (Mass media) 6. Others, specify________ |
| 206 | Can tetracycline cure all disease? | 1. Yes 2. No |
| 207 | Can amoxicillin cure common cold? | 1. Yes 2. No |
| 208 | Can ciprofloxacin cure all types of diarrhea? | 1. Yes 2. No |
| 209 | Can antibiotics prepared for human be used to animals? | 1.Yes  2. No |

**Part III. Common antibiotics use related questions**

| **SNo** | **Variables** | **Response** | **Remark** |
| --- | --- | --- | --- |
| 301 | Have you taken any antibiotic in the last one year? | 1. Yes 2. No |  |
| 302 | Which antibiotic you used? | 1. Amoxicillin 2. Ampicillin 3. Tetracycline 4. Ciprofloxacin 5. Chloramphenicol 6. Metronidazole 7. Doxycycline 8. Other specify_______ | If yes to Q 301 |
| 303 | To whom you took the drug | 1. To myself 2. To family Member | If yes to Q 301 |
| 304 | For what health problem you took the drug(s)? | 1. Cough 2. Diarrhea 3. Fever 4. Respiratory system symptoms 5. Injury/Wound 6. Urinary tract symptooms 7. Headache 8. Colic 9. Others, specify ____________ | If yes to Q 301 |
| 305 | Where did you get the drug? | 1. Examined/consult health care professional and prescribed by him/her 2. Directly bought from pharmacy 3. Lend from other family member, neighbor. 4. Bought from non-pharmacy source | If yes to Q 301 |
| 306 | Did you discontinue therapy once your symptoms subside? | 1. Yes 2. No |  |
| 307 | Do you keep leftovers antibiotics for future use? | 1. Yes 2. No |  |
| 308 | How often do you use non-prescribed antibiotics? | 1. Never 2. Rarely 3. Often 4. Very often |  |
